# A Prospective, Observational Study Evaluating the Impact and Safety of an Emergency Physician Collaboration With a Nurse Triage Phone Line

**DOI:** 10.1016/j.acepjo.2026.100424

**Published:** 2026-06-02

**Authors:** Elizabeth C. Fogelson, Christopher S. Russi, Asif Iqbal, James M. Colletti, Gregory M. Davis, Rachel A. Lindor, David M. Nestler, John C. Schupbach, Laura E. Walker, Derick D. Jones

**Affiliations:** 1 Emergency Medicine, Mayo Clinic Health System, Albert Lea and Austin, Minnesota, USA; 2 Department of Emergency Medicine, Mayo Clinic, Rochester, Minnesota, USA

**Keywords:** emergency medicine, telehealth, triage, clinical decision support

## Abstract

**Study Objective:**

Nurse triage phone lines (NTPLs) are widely used to guide patients to appropriate care levels, often relying on conservative clinical decision support systems (CDSS). This study describes the association of integrating emergency medicine physician consultation (TeleEM) with NTPL recommendations, and subsequent emergency department (ED) utilization.

**Methods:**

From October 2020 to July 2024, a prospective, observational study was conducted across a multistate health system. TeleEM physicians reviewed NTPL cases for which nurse staff requested consultation and could agree with, upgrade, or downgrade the CDSS recommendation. Seventy-two–hour ED utilization following TeleEM consultation was also assessed.

**Results:**

Among 15,196 TeleEM consults, physicians agreed with the CDSS in 56% of cases, downgraded to a less urgent endpoint in 34% of cases (n = 5164), and upgraded to a more urgent endpoint in 10% of cases (n = 1517). Of downgraded patients, 32.1% (n = 1656) presented to the ED within 72 hours, and 92.2% of those were discharged from the ED. Of upgraded patients, 72.7% (n = 1,103) presented to the ED within 72 hours, and 88.5% of those were discharged from the ED. The most common ED chief complaints among downgraded patients who presented within 72 hours were chest pain (12.9%), abdominal pain (7.0%), and dizziness (4.6%).

**Conclusions:**

Integrating emergency physician input into NTPL calls was associated with changes in triage disposition in >40% of cases. Among patients whose recommendations were downgraded, lower observed ED utilization was seen relative to patients whose recommendations were upgraded, although nearly one-third of downgraded patients still presented to the ED within 72 hours. These findings should be interpreted as associative rather than causal.

**The Bottom Line**

Can adding an emergency physician to nurse triage phone calls change how patients use emergency departments (EDs)? In this large observational study of more than 15,000 consultations, physician input changed the recommended level of care in 44% of cases. Among patients whose care was downgraded, 32% still visited the ED within 72 hours, and most were discharged. These findings suggest that physician-supported triage is associated with lower observed ED use for some patients but does not eliminate subsequent visits. Further study is needed to evaluate safety outcomes and determine whether this approach causally changes ED utilization.

## 1 Introduction

### 1.1 Background

Emergency departments (EDs) serve as the safety net for the United States’ health care system, providing care every day at all hours. The complications of serving this role include needing to accommodate varying acuities at presentation and unexpected surges in volume, both of which contribute to prolonged wait times and potential overcrowding.

Contributors to ED crowding have been divided into 3 components: input, throughput, and output.^1^ Mismatch among these components and subsequent overcrowding creates potential adverse effects, including increased length of stay, delays in patient care, worse clinical outcomes, and higher mortality.^2^ Most efforts to address this mismatch have focused on throughput and output, including smoother intra-ED operations and expedited dispositions.^3^^,^^4^ Attempts to address ED input have been more limited but have included identifying sources of ED referrals and creation of alternative care sites for lower acuity patients.^5^ Increasingly, models incorporating use of telehealth to assist with pre-ED triage have been described with encouraging outcomes.^6^^,^^7^

One model for refining ED input includes nurse triage phone lines (NTPLs), which are services often provided by health care systems and insurance companies to direct patients to the right level of care.^8^ The phone lines generally use a clinical decision support system (CDSS) as a screening instrument to direct patients to self-care, emergency care, or somewhere between those 2 ends of the spectrum, depending on community resources. These screening tools are designed to be conservative and may direct patients to seek emergency care when outpatient care could suffice.^8^

A limited number of studies have examined the effect of offering a second level of triage in which the nursing staff on the NTPL are able to engage with emergency physicians by telemedicine (TeleEM) to augment the guidance of the CDSS. The results from these studies are promising but limited by their narrow focus on veteran and pediatric populations.^9^^,^^10^ The ability of a physician-augmented NTPL to influence subsequent ED utilization in a general community population remains unknown.

### 1.2 Importance

Reducing avoidable ED visits is paramount to addressing the issue of ED overcrowding and maintaining the ability to provide high-quality care. If TeleEM consultation is associated with lower ED utilization by redirecting some patients to alternate care settings more effectively than NTPL alone, this strategy could have benefits for both patients and health systems.

### 1.3 Goals of This Investigation

We aimed to describe the impact and outcomes of TeleEM to augment the NTPL process within a multistate health system. We hypothesized that use of the TeleEM physician in this role would be associated with lower observed ED utilization among some patients.

### 2 Methods

#### 2.1 Study Design and Setting

We performed a prospective, observational cohort study following all patients who contacted a NTPL from October 2020 to July 2024 and evaluated all calls in which the TeleEM physician was contacted. Patients were followed for 72 hours to capture subsequent health care utilization within the health care system.

Nurses staffing the phone line had the option of consulting an emergency physician via telemedicine at any time between 7 a.m. to 1 a.m. to obtain a second opinion regarding disposition recommendations. For their initial recommendations, the triage nurses rely on a CDSS—the Schmitt-Thompson Clinical Content algorithm^11^—with endpoint recommendations divided into 4 main categories: (1) seek immediate care, (2) see a health care provider within 12 to 24 hours, (3) see a provider within 3 days, or (4) home care. The “seek immediate care” category is further divided into 5 subcategories: (1) call 911 now, (2) seek immediate care, (3) go to ED now, (4) contact a health care provider within a few minutes, and (5) see a health care provider within 4 hours.

Patients using the NTPL were part of a large health system in the Midwest including one academic medical center in Minnesota (MN) and a combination of 16 critical access and community hospitals located across southern Minnesota and western Wisconsin. The NTPL started at the community hospitals in 2013 and expanded to include the academic site’s patients in 2023. It currently handles >400,000 calls annually.

### 2.2 Selection of Participants

All recorded TeleEM consultations from the NTPL during the study period were included. TeleEM consultation was nurse-initiated and not required for all NTPL calls. Any cases in which a discussion occurred but the NTPL did not record the TeleEM physician’s recommendation were excluded. Patients were followed using their medical record number to assess any additional health care utilization.

### 2.3 Interventions

Beginning in October 2020, nurses staffing the NTPL were encouraged to contact the TeleEM physician when the CDSS recommendations fell into the “seek immediate care” category. In these cases, the NTPL nurse would call the TeleEM physician, obtain a clinical recommendation, and record the physician’s recommendation in the call log that is embedded in the electronic medical record. In some cases, physicians would ask to speak to the patients directly to guide their recommendation, but this was not common. TeleEM physicians were emergency physicians located at a tertiary care center in the health system, and recommendations were based on clinical judgment rather than a standardized protocol.

### 2.4 Measurements

We tracked the total number of calls between the triage nurse and TeleEM, the original NTPL recommendation, and the TeleEM physician’s recommendation to either maintain, expedite, or downgrade the disposition.

We followed all included patients to identify the occurrence of an ED visit at 1 of the 19 affiliated EDs within 3 prespecified intervals after the initial phone consultation: (1) within 4 hours; (2) within 24 hours; and (3) within 72 hours. We evaluated the ED disposition of patients who were not advised to by the TeleEM physician to undergo urgent ED evaluation but who still presented to an ED within 72 hours.

Finally, we also captured data on patient demographics (age, sex, gender, language, race, ethnicity, insurance status) and ED encounter characteristics (acuity level, chief complaint, means of arrival, diagnosis, ED disposition). Sex was defined based on the binary sex variable recorded in the electronic medical record, while gender identity reflects patient-reported gender identity when available in the medical record. Data were collected from the electronic medical record (Epic; Verona, WI).

### 2.5 Outcomes

Outcome measures included frequency of TeleEM physician disagreement with the NTPL recommendation and subsequent ED utilization within 72 hours after TeleEM involvement. We also evaluated the number of patients who did present to the ED urgently when it was recommended that they do so, recording ED presentation within 4, 24, and 72 hours of the TeleEM call, thereby creating a measure of recommendation adherence. Because of the observational design, these outcomes were intended to describe utilization patterns following TeleEM consultation rather than estimate causal effects.

### 2.6 Data Analysis

Simple descriptive statistics were used to demonstrate proportions. Continuous features are summarized with means, and standard deviation, and categoric features are summarized with frequency counts and percentages. Sample size was determined as a convenience sample based on the duration of the study. TeleEM providers were not blinded to the study hypothesis.

Comparisons in demographic and ED encounter characteristics were analyzed for differences in the patients, where there was and was not agreement with the nurse triage CDSS endpoint. Cases where there was agreement with the triage CDSS endpoint were compared with cases where the TeleEM physician disagreed with the CDSS endpoint. Chi-square test statistics and *P* values were calculated for differences in categoric variables, and analysis of variance F-statistic with *P* values were calculated for differences in continuous variables.

### 2.7 Ethics Approval

The protocol was approved by the institutional review board.

## 3 Results

### 3.1 Participants

During the study period, 16,594 calls were placed between the NTPL and the TeleEM physician. Of these, 15,196 calls (91.6%) included documented physician recommendations and were included in the analysis. There were 13,917 unique patients with a median age of 42.0 years (43 years for females, 41 years for males). Most of the patients were female (57.9%) and White (93.2%), whereas 4.3% identified their ethnicity as Hispanic or Latino, 1.3% identified as African American, and the remainder of patients were represented at less than 1% of the subject population.

### 3.2 Main Results

#### 3.2.1 Initial triage recommendations

**Figure 1:**
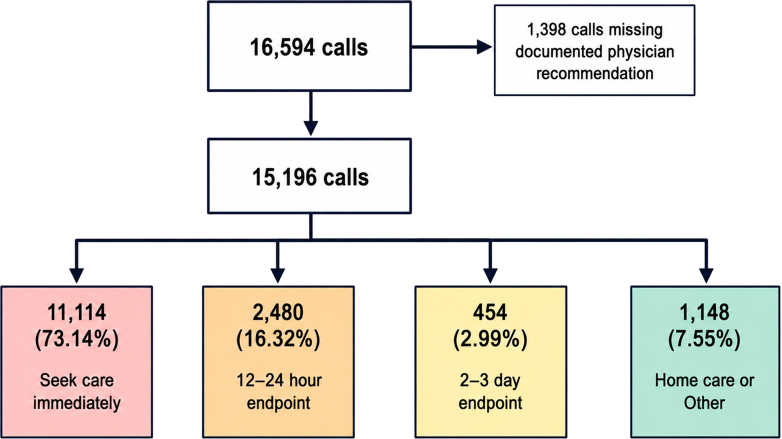
Initial NTPL CDSS endpoint recommendations among included TeleEM consultations. Percentages are calculated out of 15,196 calls with documented physician recommendations.

Before the telemedicine physician input, the NTPL CDSS recommended: “Seek Immediate Care” (n = 11,114), “See a health care provider within 12 to 24 hours” (n = 2480), “Home Care or Other” (n = 1148), or “See a health care provider within 2 to 3 days” (n = 454). (Fig 1)

**Figure 2:**
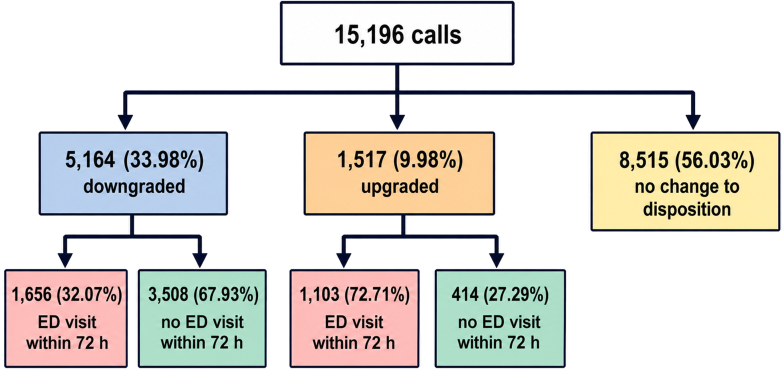
Changes in disposition based on telemedicine physician consult and subsequent emergency department utilization within 72 hours. Percentages are calculated out of 15,196 calls with documented physician recommendations.

Of the 15,196 nurse calls analyzed, the telemedicine physician agreed with the NTPL CDSS recommendation in 8515 cases (56.0%). The telemedicine physician downgraded the urgency in 5,164 (34.0%) and upgraded urgency in 1517 cases (10.0%). (Fig 2)

### 3.2.2 ED utilization

Overall, a total of 8954 patients (58.9%) presented to the ED within 72 hours of their NTPL call.

Of the patients upgraded to a more urgent endpoint, 72.7% (n = 1103 of 1517) presented to the ED within 72 hours. Of those who presented, 88.5% were discharged from the ED, whereas the remainder were admitted, placed in observation, transferred, or left before completion of care. The most common chief complaints in this group were abdominal pain (8.9%), fever (5.6%), and fall (3.7%). The most frequent ED discharge diagnoses among this group of patients were abdominal pain (3.9%), chest pain/atypical chest pain (3.6%), and viral syndrome (2.8%).

Among patients who were downgraded to a less urgent endpoint, 32.1% (n = 1656 of 5164) presented to the ED within 72 hours. Of those who presented, 92.2% were discharged from the ED, whereas the remainder were admitted, placed in observation, transferred, or left before completion of care. The most common ED chief complaints were chest pain (12.9%), abdominal pain (7.0%), and dizziness (4.6%). The most common ED discharge diagnoses among these patients were chest pain/atypical chest pain (8.2%), abdominal pain (4.58%), and headache (2.0%).

### 3.3 Patient Characteristics by Disposition Change

#### 3.3.1 Agreement vs nonagreement (combined downgraded and upgraded) among patients presenting to the ED within 72 hours

Patients for whom the physician disagreed with the original NTPL CDSS recommendation were more likely to present to the ED with a chief complaint of abdominal pain, chest pain, fall, or fever. These patients were more likely to be younger, female, or speak a language other than English. There were no significant differences in acuity, final disposition, financial class, payor, ethnicity, gender identity, or race. (Tables 1 and 2,^1^)

**Table 1:** Demographics for patients who presented to the ED after calling the Nurse Phone Triage Line.^a^

Demographic	Category	Agree (N = 5705)^b^	Disagree^b^ (N = 2759)	Upgrade (N = 1103)	Downgrade (N = 1656)
Financial class	Commercial	1883	882	359	523
	Employee	493	244	101	143
	Medicare Advantage	691	284	116	168
	Government	2513	764	454	663
	Subtotal	5580	2174	1030	1497
	Total	7754		2527	
Ethnicity	Not Hispanic or Latino	5830	2590	1045	1545
	Hispanic or Latino	285	138	46	92
	Choose not to disclose	45	22	7	15
	Unknown	12	3		2
	Subtotal	6172	2753	1098	1654
	Total	8925		2752	
Gender identity^c^	Female	2751	1272	486	786
	Male	1720	698	279	419
	Nonbinary or genderqueer	19	11	1	10
	Choose not to disclose	15	8	1	7
	Transgender male	6	2	1	1
	Subtotal	4511	1991	768	1223
	Total	6502		1991	
Language	English	6136	2716	1090	1626
	Spanish	32	23	3	1
	Somali	3	1	0	20
	Other	8	10	6	5
	Subtotal	6179	2750	1099	1652
	Total	8929		2751	
Sex^c^	Female	3570	1655	650	1005
	Male	2625	1104	453	651
	Subtotal	6195	2759	1103	1656
	Total	8954		2759	
Race	White	5836	2605	1042	1563
	African American	126	65	25	40
	Asian	27	24	10	14
	Other	36	17	9	8
	Choose not to disclose	44	14	5	9
	Subtotal	6069	2725	1091	1634
	Total	8794		2725	
Mean age at arrival (y)		44.18 (28.43)	42.59 (27.88)	42.3 (29.4)	42.78 (26.83)

ED, emergency department.

a Totals may be higher or lower than actual number of presentations due to multiple selections per category and missing data.

b The “Disagree” category is the combined total of both “Upgrade” and “Downgrade” groups.

c Sex was derived from the demographic sex field in the electronic medical record. Gender identity reflects patient-reported gender identity when documented.

**Table 2:** ED Visit characteristics for patients who presented to the ED after calling the Nurse Phone Triage Line.^a^

Variable	Category	Agree (ED visit) N = 5705^b^		Disagree (ED visit) (N = 2759)		Upgrade (N = 1103)		Downgrade (N = 1615) (%)	
Means of arrival	Ambulance	167	2.80%	47	1.76%	18	1.63%	29	1.80%
	Carried	489	8.21%	210	7.88%	105	9.52%	105	6.50%
	Other	0	0.00%	0	0.00%	19	1.72%	0	
	Personal vehicle	498	8.36%	245	9.20%	95	8.61%	150	9.29%
	Walk-In	4264	71.56%	1941	72.86%	751	68.09%	1190	73.68%
	Wheelchair	541	9.08%	221	8.30%	93	8.43%	128	7.93%
	Total^1^	5959		2664					
Acuity (ESI)	1	1	0.02%	1	0.04%	1	0.09%	0	0.00%
	2	356	6.24%	160	6.36%	58	5.26%	102	6.32%
	3	3774	66.15%	1647	65.49%	639	57.93%	1035	64.09%
	4	1434	25.14%	640	25.45%	293	26.56%	345	21.36%
	5	140	2.45%	67	2.66%	25	2.27%	42	2.60%
	Total	5705		2515					
ED chief concern	Chest pain	499	8.75%	231	8.67%	23	2.09%	208	12.88%
	Abdominal pain	471	8.26%	211	7.92%	98	8.88%	113	7.00%
	Shortness of breath	336	5.89%	97	3.64%	33	2.99%	64	3.96%
	Fever	278	4.87%	132	4.95%	62	5.62%	70	4.33%
	Dizziness	213	3.73%	88	3.30%	42	3.81%	74	4.58%
	Fall	188	3.30%	98	3.68%	49	4.44%	46	2.85%
	Vomiting	0	0.00%	0	0.00%	40	3.63%	0	0.00%
	Assumed total^b^	5705		2664					
ED disposition	Admit	247	4.02%	85	3.11%	42	3.81%	43	2.66%
	Discharge	5448	88.72%	2465	90.13%	976	88.49%	1489	92.20%
	Hospital observation	190	0.31%	93	3.40%	38	3.45%	55	3.41%
	Left without being seen	95	1.55%	36	1.32%	16	1.45%	20	1.24%
	Transfer to health care facility	161	2.62%	56	2.05%	23	2.09%	33	2.04%
	Total^a^	6141		2735					
ED primary diagnosis	Pain chest/atypical chest pain	312	5.47%	150	5.44%	40	3.63%	133	8.24%
	Abdominal pain	253	4.43%	117	4.24%	43	3.90%	74	4.58%
	Nausea and vomiting	126	2.21%	43	1.56%	25	2.27%	18	1.11%
	Injury head initial	112	1.96%	55	1.99%	27	2.45%	0	0.00%
	Headache unspecified	131	2.30%	48	1.74%	16	1.45%	32	1.98%
	Viral syndrome	99	1.74%	60	2.17%	31	2.81%	29	1.80%
	COVID-19	150	2.63%	34	1.23%	9	0.82%	25	1.55%
	Assumed total^b^	5705							

ESI, Emergency Severity Index; ED, emergency department.

a Numbers may not be consistent with the true number of presentations due to multiple values being present for a single patient.

b Acuity total used to estimate total number of presentations because each patient should be assigned only one acuity level.

#### 3.3.2 Agreement vs downgraded

Downgraded patients differed significantly in chief complaint, ED diagnosis, disposition, arrival method, gender identity, and race. Downgraded cases showed higher rates of headache and chest pain diagnoses but fewer abdominal pain or COVID diagnoses. They were less likely to arrive by ambulance, less likely to be admitted, and more frequently discharged. This group of patients were more often female or nonbinary, and more likely to be African American. (Tables 1 and 2,^1^)

#### 3.3.3 Agreement vs upgraded

Upgraded patients differed significantly in chief complaint, ED diagnosis, language, and age. These patients were more likely to report abdominal pain and head injuries, and less likely to report chest pain, COVID, or headache. They tended to be younger and less likely to speak English. (Tables 1 and 2,^1^)

## 4 Limitations

This study has several limitations of note. We expect that there is an unknown amount of variation among physicians who provide care via our telemedicine program. We have few guidelines or protocols to standardize recommendations, and it is largely left to physician discretion. We have varying levels of risk tolerance and comfort with telemedicine practice among our physicians.

TeleEM consultation was nurse-initiated and not universal across all NTPL calls, which introduces the possibility of selection bias and confounding by indication. Because consultation was requested based on nurse judgment and clinical context, patients who received TeleEM input may have differed systematically from NTPL callers who did not.

The clinical setting contributes additional limitations due to hours of service for outpatient care. When CDSS endpoints indicated that the patient should be seen within 4 hours and the clinic would be closed before this time, the physician would have a different situation than if the clinic were open longer and had additional appointments. These patients who call during the hours in which the clinic is closed or unavailable would either need to be upgraded to an ED visit or have their recommended visit time extended.

An additional variable we could not control is the decision making of the clinicians who cared for the patient after their arrival to the ED. Their discretion determined the disposition of the patient, and we did not investigate if TeleEM involvement before ED arrival changed the decision making or behaviors. Reasons for the admission and decision making process were not characterized.

The study was based on medical record review. It is possible that patients presented outside of the health system included in our medical records, and that our results under-report the ED and hospital utilization of these patients. In addition, our medical record more consistently captured binary sex variables than patient-reported gender identity. Sex data were derived from the demographic sex field within the electronic medical record, while gender identity data were available only when documented separately. As such, our ability to fully assess gender identity within the study population was limited.

A total of 1398 calls (8.4%) were excluded because physician recommendations were not documented. These excluded cases may have differed systematically from included cases, and this may have introduced bias. Additionally, our data set did not include NTPL calls that did not result in a TeleEM consult, so we are not able to compare characteristics between these groups.

Finally, the study was not designed with an a priori effect size, lowering its scientific rigor. In addition, this study did not evaluate serious adverse outcomes such as intensive care unit admission, death, missed diagnoses, or delayed diagnosis, limiting conclusions regarding safety.

## 5 Discussion

Our findings suggest that integrating an emergency telemedicine physician into the traditional protocol-driven nurse triage line was associated with substantial changes in disposition recommendations. Among the patients whose disposition was downgraded, 32% presented to the ED within 72 hours compared with 73% of those whose acuity was upgraded by the physician. These results support the role of a telemedicine physician as a potentially valuable addition to the traditional nurse triage model, while remaining associative rather than causal.

Chest pain and abdominal pain were the most common symptoms associated with a change in disposition recommendation by the telemedicine physician. Without physician input these patients would have all been conservatively directed to the ED. Physician involvement in triage may allow for more nuanced assessment and redirection of some patients toward lower acuity care settings. This highlights the value of physician clinical judgment in augmenting an intentionally conservative nurse-triage screening algorithm, especially when evaluating potentially high-risk chief complaints such as chest pain and abdominal pain.

Importantly, approximately one-third of patients whose disposition was downgraded by the TeleEM physician still presented to the ED within 72 hours. This finding suggests that downgrading does not equate to avoidance of ED visits, and the magnitude of any reduction in ED utilization should be interpreted cautiously. Patient decision making, symptom progression, access barriers, and individual risk tolerance may all have contributed to subsequent ED presentation despite the TeleEM recommendation.

Our demographic analysis of NTPL showed 57.9% of patients were female and 93.2% identified as White. This does not reflect the demographics of the region based on the census information from the county where our largest medical center is located. This suggests unequal access to the NTPL and underscores the need to ensure equitable access for all patients.

Although the high ED discharge rate among downgraded patients is reassuring, our study did not directly assess clinical safety outcomes. We did not evaluate adverse events such as missed diagnoses, ICU admission, death, or delays in diagnosis. Future work should more directly assess these outcomes to determine the safety of this model.

Cost-effectiveness was also out of scope of this study but remains a key area of focus for future research evaluating the benefits of telehealth interventions. There is an opportunity when directing patients to “right care, right place, right time” to decrease the total cost of care. Quantifying these potential cost savings is challenging and will require dedicated economic analysis.

Important factors that played into our success include access to alternate care locations, a coordinated system of care in our region, multispecialty collaboration, and a unified electronic health record. After hours, if a patient needs to be seen acutely, the only course of action remains an ED visit or an urgent care visit. Other health systems may have additional advantages or limitations in terms of access to expedited outpatient appointments or diagnostics that may necessitate an ED visit. The ability to defer ED visits to primary care has economic impacts, particularly for organizations that participate in an Accountable Care Organization or self-insure their employees’ health care, as well as to the patient directly.

In conclusion, this project demonstrates the potential role of a virtual emergency physician presence in influencing triage recommendations and subsequent patterns of ED utilization. Patients who received additional advice from TeleEM often followed that advice, and when they did present to an ED after a downgraded recommendation, most did not require hospitalization. However, these findings should be interpreted as descriptive and associative rather than causal. In an era of persistent ED overcrowding, this highlights an alternative pathway for emergency physicians to contribute to system-level care navigation and resource stewardship.

## Author Contributions

EC contributed to study design, data interpretation, manuscript drafting, and coordination of manuscript revisions. CSR contributed to study conception, study design, and critical revision of the manuscript. DDJ contributed to project oversight, data acquisition, statistical analysis, data interpretation, and critical revision of the manuscript. RAL contributed to project administration, project oversight, mentorship, and critical revision of the manuscript. JMC contributed to study conceptualization, development of the introduction, mentorship, operational leadership, and critical revision of the manuscript. AI contributed to operational implementation and administrative support. LEW contributed to study conceptualization, manuscript development, data interpretation, mentorship, and critical revision of the manuscript. JCS contributed to literature review, citation management, and critical revision of the manuscript. GMD and DMN contributed to data interpretation and critical revision of the manuscript.

## Funding and Support

By *JACEP Open* policy, all authors are required to disclose any and all commercial, financial, and other relationships in any way related to the subject of this article as per ICMJE conflict of interest guidelines (see www.icmje.org). The authors have stated that no such relationships exist.

## Conflict of Interest

All authors have affirmed they have no conflicts of interest to declare.
